# Round the Hospitals

**Published:** 1917-09-29

**Authors:** 


### ROUND THE HOSPITALS.

The Queen, when in France, spent much time in visiting warrior patients in the hospitals. The gracious interest which Her Majesty took in the warriors was most gratefully welcomed by the patients. Never before has a reigning monarch of England had such an opportunity of meeting, in their hours of trial, more representative groups of suffering fellow-subjects than those afforded by wards "somewhere in France." In wards containing some eighty patients there were representatives of nearly every county in England, and of many parts of Wales, Scotland, Ireland, Australia, the Channel Islands, and the Isle of Man. Practically every English county which sent its warriors to the war had a representative in these wards. We could wish that the people at home, and especially the pessimists, the do-nothings, if any there still be in the British Isles, could be brought to realise the teachings of a visit to hospital wards containing our injured warriors. Welcome as the Queen's presence in these wards proved to their inmates, we make bold to think that Her Majesty's experiences during these visits will bear fruit, and carry with them remembrances which will be among the most precious memories of her life.

Another most welcome hospital visitor is H.R.H. the Princess Louise, who, with great generosity, has often lightened the trouble and relieved the anxieties and sufferings of sick nurses over the water. Her Royal Highness is untiring in her visits to the hospitals, where she makes it a practice to converse with each individual patient in every ward she enters. Hospital workers realise that patients who suffer from severe fractures and injuries to joints, entailing confinement in bed for many weeks in all sorts of postures, which modern science and skilled surgeons, especially since the war, have rendered relatively endurable, and fruitful in the saving of limbs which at the outset seemed beyond hope of recovery, are helped by the presence of sympathetic visitors to an extent hardly appreciated by the outside public. No wonder that a visit from H.R.H. Princess Louise is looked forward to with joyous appreciation.

---

"Somewhere in France" a number of members of the W.A.A.C., the latest example of woman's patriotism and devotion, give up much of their limited leisure on Sundays, after the hard and

☛ Previous articles appeared in The Hospital of August 4, 11, 18, 25, and September 1, 15, and 22.

**ROUND THE HOSPITALS**—*(continued).*

continuous work of the week, to visiting the patients in a hospital. It has been our privilege to meet them while so engaged, and to have had an opportunity of testing the results of their womanly forethought. A soldier is nothing if not gallant, but the joy and happiness derived from such visitors, which help to remind him of home, to make the hours pass without monotony, and revive welcome remembrances, cannot be exaggerated. They are the latest evidence of the enormous force the great war has exercised in bringing all classes together and uniting all in work and friendly acts which forecast a happier and better world, when freedom is restored to all free peoples, and they have at last won by their valour and self-sacrifice the right to live their own lives and pursue their own peaceful occupations at their will and pleasure. Every hospital worker will appreciate the act of a convalescent warrior patient helping in a large ward at tea-time. Whenever he found a patient ill enough to hesitate as to whether he would take one or two of some very appetising sandwiches of potted meat, he encouraged and cheered each man with the remark: "Take one, and if you'd like another after eating this I'll bring one to you gladly." Yet some women hold that men have no gifts for nursing.

---

Sir Henry Lucy has sent to the *Times* an officer's description of flowers at the Front, which faithfully portrays the love our warriors have for flowers of any kind, for they change the whole atmosphere and bring thoughts of home. He describes how he found at Fampoux, between piles of bricks, shell-holes, dirt, and every sort of débris, a rose in full bloom and a lily wafting its delicious scent; and as he met each blossoming flower, his imagination caused him to realise how this unhappy village looked before the war. The central attraction amongst innumerable shell-holes was a small patch of ground absolutely carpeted with buttercups, over which blazed bright red poppies intermixed with the bluest of cornflowers—a really glorious corner which quickly brought memories of home.

---

Cheerfulness in the sick is the high-road to recovery, as we have often insisted. Nowhere is the evidence of this truth more forcibly illustrated than in the wards crowded with wounded warriors, whose joyous faces under the most trying of circumstances win for them the admiration—sometimes, we fancy, the envy—of non-combatants of both sexes. Most matrons love flowers, and some of the most striking arrangements of them in vases are to be found in the matron's sanctum. Somewhere in France a distinguished Australian sister-matron, who has won more than one medal in previous wars, is famous for the flowers she invariably attracts and keeps near her. One day a terrible case of injuries came into the hospital, which moved her compassionate heart and called forth all her sympathy. When the poor fellow had been attended to by the surgeons and placed in a bed, she visited the ward, taking a selection of flowers with her. Her advent was welcomed by those who were comparatively convalescent, and the presence of the flowers brought a smile to the face of the injured man, who forgot his suffering for the moment in the gladness he experienced and the joy with which he received some flowers to be kept constantly near him by his bedside. Our warriors for the most part have wonderful constitutions, and the rapidity with which they rally from the shock imposed upon them by the hideous wounds, caused by shrapnel and other abominable things used in the present war, is a remarkable and welcome fact. In the case of the patient just mentioned, the effect of the flowers was remarkable, having regard to the nature of his injuries. It is gratifying to know that the flowers not only brought immediate consolation and help, but had much to do with this patient's steady recovery. Now that we have some real summer weather, after the attempts at sunshine—mostly nebulous—which have characterised many days of 1917, we hope all who have gardens will, as many do already, remember the hospitals, the wounded, and their love of flowers, and send regularly the best they can give. Then the flowers of 1917 may yet do better service for British warriors than they have ever before had an equal opportunity to yield.

---

Now that the question of flowers has come forcibly to the front, it may be well to point out that some of the most attractive of the spacious wards in British hospitals are those which have fine large tables placed at the end of the ward near the door of entrance. These tables seem to have a natural attraction for flowers, and their appearance, from this circumstance, is the first feature to compel notice and help to cheer the inmates of the ward. The wards of the Gloucester Royal Infirmary are famous for the splendid tables they contain. These have been made out of the timber of famous battleships of the past. The way in which they are kept, and their state of preservation, are so attractive as never to be forgotten by the observant visitor. It would not be a bad idea, as a memento of the Great War, for some wealthy resident in each county and large city to seize the privilege of presenting some tables of this kind, made from the oak of British ships, to the hospitals with which they are connected. We should like to have an opportunity of giving a prize to the sister whose ward contained the most attractive table of the character we have in view. If our readers favour the idea, we shall no doubt hear from them and receive good photographs in due course.

---

The present are undoubtedly women's days. The war has moved the women of all classes and of most nations to come forward and render service of the highest value—service, too, which is more often than not accompanied by the exercise of self-denial, with hard, continuous work which, from its volume and quality, has surprised the nations. In England, and even in London, to the no small

**ROUND THE HOSPITALS**—(*concluded*).

discredit of those responsible, British nurses have been called upon to occupy unsuitable accommodation, and to suffer conditions of service which ought never to have been permitted. Originally they entered such service in good faith as their contribution to the war, but the plea that in war-time "roughing it" was a virtue rendered necessary by economical considerations is usually as ill-founded as it is discreditable to those who advance it. "Somewhere in France" the conditions for nurses are not always ideal, but the American nurses in one large hospital at least have had planned and built for them a series of large pavilions, arranged within a stockade, which are as picturesque and attractive to the eye as they are comfortable and inspiriting to the occupants. Probably few attempts to house nurses under the most suitable conditions have been better fulfilled, or are more likely to constitute a model for future adoption than the one we have in mind.

In view of the recent destruction of windows at a certain hospital in London during an air-raid, it may be useful to note that in a large hospital run by one of our Allies on the Western Front—whose bombardment is not infrequent—tissue paper is pasted over each window with a view to its protection. The tissue paper is sufficiently transparent to admit the necessary light. A sister who has been several months at the hospital in question vouches for the usefulness of the method in the case of the windows there. She found that a bomb might shake the whole hospital, but the windows—of course, when not directly struck—remained intact. Most persons know of this method for packing glazed pictures in order to save the glass from being broken during transit.

The shortage of trained nurses is likely to lower the standard of nursing in small workhouse infirmaries. The Guardians of Ruthin Union have decided to advertise for "an experienced, though not necessarily certificated nurse" to take charge of their workhouse sick-wards. It is probably true that a partially trained woman would do the ordinary work of such a post, if she had skilled help in any emergency, and if she were the right type of woman. But if certificated nurses are hard to get, the excellent uncertificated ones are still more rare. They were fairly plentiful thirty years ago; but now there is a higher standard of thoroughness in women's work, as well as better opportunities of training. The type of woman who has the qualities necessary for a really good nurse will take her full training and receive a proper certificate. The exceptions to this rule are those who through ill-health or family troubles have been unable to complete their training. Perhaps the Ruthin Guardians will succeed in obtaining one of these.

The remark of one Guardian, that "a girl" at a salary of £20 formerly did all the nursing, gives rise to one or two reflections. No one "girl"—no one woman even—can nurse a ward of bedridden patients (apparently both night and day) properly. The work is heavy, monotonous, and often extremely trying. If the nurse is conscientious, she gets no proper rest or recreation. If she is not so, the patients are neglected. With regard to the salary, it can only be hoped that the arrangement came to an end because no woman could be found who would allow her labour to be so disgracefully sweated.

Contract prices have gone up in some instances to a level quite unjustifiable under existing circumstances, and the excuse is the natural one that the contractor aims in making his tender at protecting himself against any further rise in prices. Under these conditions it is of doubtful advantage to contract, and we note that the York Guardians recently instructed their Provisions Committee to purchase direct from tradesmen in the city, instead of by tender. In York during the year April 1916 to April 1917 beef rose per stone from 9s. 7½d. to 15s. 9d.; mutton from 11s. 11½d. to 16s. 4d.; flour from 44s. a sack to 59s. 6d. Margarine, which used to be 56s. a cwt., was quoted for at 100s. a cwt.; cheese, formerly 108s. a cwt., was quoted at 186s.; and bacon, formerly 102s., was quoted at 168s. These figures illustrate the difficulties which beset purchasers placing large orders, and managers in our judgment do wisely in refusing to bind themselves to contractors who are themselves subject to panic in the present crisis. Where possible, recourse should be had to the open market.

Polling one-third of the total votes cast for the twenty-one candidates, Miss R. C. McAdams, a Canadian nursing sister now attached to the Orpington Military Hospital in this country, has been elected to the Alberta Parliament as one of the two representatives allocated to the Canadian soldiers and nurses serving in France and England. She thus becomes the second woman member of a Canadian Legislature, but is probably the first nurse in the world to be elected an "M.P." Sister McAdams, who is a dietetic expert attached to the Canadian A.M.C. for the oversight of the feeding of the Dominion's fighting forces, is a sister-in-law of the Canadian Food Controller.

☞ It is our invariable practice to pay for all items of news which we may publish. The minimum payment is 5s. Paragraphs of about twenty lines in length—that is to say, of 150 words or less—are preferred. In this section during the war we propose to give 10s. to the correspondent who may send us in any week the best incident connected with hospital life and work. In this way we hope to afford to all practical aid and encouragement; while those who reciprocate will be able to render us invaluable service. Contributions should be addressed to The Editor, The Hospital, 28 and 29 Southampton Street, Strand, London, W.C., and, to be in time for the current week's issue, should reach him by the first post on Mondays.

For "Nursing Affairs in Ireland," see p. 532; An Institution Food Controller, p. 536; Smoking in Hospital, p. 537; Matrons' and Sisters' Appointments, p. 538.

